# CNT Foam-Embedded Micro Gas Preconcentrator for Low-Concentration Ethane Measurements

**DOI:** 10.3390/s18051547

**Published:** 2018-05-14

**Authors:** Janghyeon Lee, Si-Hyung Lim

**Affiliations:** 1 Department of Mechanics and Design, Kookmin University, Seoul 136-702, Korea; leejjanghyun@naver.com; 2 School of Mechanical Engineering, Kookmin University, Seoul 136-702, Korea

**Keywords:** breath analysis, ethane, micro gas preconcentrator, gas-adsorbing materials, carbon nanotube foam

## Abstract

Breath analysis has become increasingly important as a noninvasive process for the clinical diagnosis of patients suffering from various diseases. Many commercial gas preconcentration instruments are already being used to overcome the detection limits of commercial gas sensors for gas concentrations which are as low as ~100 ppb in exhaled breath. However, commercial instruments are large and expensive, and they require high power consumption and intensive maintenance. In the proposed study, a micro gas preconcentrator (μ-PC) filled with a carbon nanotube (CNT) foam as an adsorbing material was designed and fabricated for the detection of low-concentration ethane, which is known to be one of the most important biomarkers related to chronic obstructive pulmonary disease (COPD) and asthma. A comparison of the performance of two gas-adsorbing materials, i.e., the proposed CNT foam and commercial adsorbing material, was performed using the developed μ-PC. The experimental results showed that the synthesized CNT foam performs better than a commercial adsorbing material owing to its lower pressure drop and greater preconcentration efficiency in the μ-PC. The present results show that the application of CNT foam-embedded μ-PC in portable breath analysis systems holds great promise.

## 1. Introduction

The presence of volatile organic compounds (VOCs) in exhaled breath has long been considered useful in the early diagnosis and monitoring of various diseases [1,2,3]. Breath analysis has been proposed as a safer and more convenient clinical diagnosis method in comparison with traditional diagnostic techniques [4]. It is a noninvasive and painless process for patients, and its sampling approach does not require medical experts [5,6,7,8,9]. The development of breath analysis has steadily progressed because of these advantages, despite the wide variations in the chemical compositions of exhaled breath and the technological challenges associated with measuring many components simultaneously [10,11]. Remarkably, some VOCs in the exhaled breath of humans are correlated to specific diseases and thus have been identified as breath biomarkers [12]. Nitric oxide (NO), carbon monoxide (CO), and several alkanes, such as ethane (C_2_H_6_) and pentane (C_5_H_12_), have been shown to be important biomarkers for various respiratory diseases [13]. In particular, the amount of ethane in exhaled breath can be used as a physiological indicator for chronic obstructive pulmonary disease (COPD) and asthma owing to its easier and faster chromatographic measurement in comparison with other hydrocarbons [14,15]. Hence, the proposed study aims to focus on exhaled ethane used as a biomarker of respiratory diseases. However, since human exhaled breath contains very low concentrations of ethane, typically in the parts per billion (ppb) range [16,17,18], accurate measurements can be difficult to achieve with conventional sensors, such as Zellpel (SyxthSense, Exeter, UK), CH-D3 (Alpha Sense, Essex, UK), and NP-13S (Nemoto, Tokyo, Japan), which have sub-ppm detection limits [19,20,21,22]. Therefore, the preconcentration of low-concentration gases during the previous stage in the commercially available gas sensors is an inevitable step for the detection and analysis of any breath biomarkers at concentrations of a few ppb or lower [23,24,25,26].

**Figure 1:**
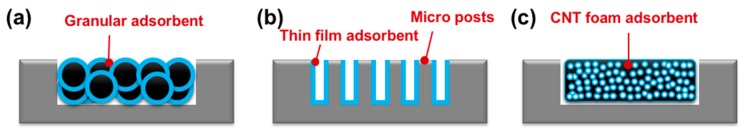
Schematic diagram showing the cross-sectional views of several different types of micro preconcentrators: (**a**) adsorbing chamber filled with granular adsorbents; (**b**) adsorbing chamber with micro-post structures coated with a thin adsorbing material; (**c**) adsorbing chamber filled with a carbon nanotube (CNT) foam as the proposed adsorbing material.

Numerous studies have been conducted with the aim of improving the sensitivities and detection limits of gas sensors using gas preconcentration, which adsorbs low-concentration, gas-phase chemicals within a specified time interval and then desorbs these using heaters within a very short time interval. Several gas preconcentration and desorption apparatuses, known as thermal desorption (TD) systems, have already been developed [27,28,29,30,31,32]. However, the existing TD systems are large, expensive instruments that consume a lot of power and require intensive maintenance [33]. Microelectromechanical system (MEMS) technology makes the miniaturization of preconcentration and desorption components possible, which can overcome these drawbacks. Several studies focusing on the fabrication of micro gas preconcentrators (μ-PC) by MEMS technology have been reported, and these can be broadly classified into two categories. They are distinguished from each other based on the chamber layout of μ-PC for gas adsorption (empty or micro-post structures) and the adsorbing material’s profile (granular or thin film). In the first type of granular adsorbing material device, channels and chambers formed on silicon substrate are filled with adsorbent beads, as shown in Figure 1a [34,35,36]. The second type of μ-PC utilizes adsorbing materials in the form of a thin film deposited on the micro-fabricated channels and chambers with micro-post structures inside, as shown in Figure 1b [37,38,39]. Both types of devices have trade-offs. The first type can provide a high sample capacity but it suffers from high pressure drops and power consumption during the preconcentration processes. The second type significantly reduces the pressure drop, though it has limited sample capacity due to having less surface area to interact with the analytes. Therefore, one of the major challenges in developing μ-PC is the synthesis of a superior adsorbing material with a high sample capacity and low-pressure drop.

In this study, highly porous and adsorbing carbon nanotube (CNT) foam was synthesized to improve the overall performances of the preconcentration devices (Figure 1c), and μ-PC filled with CNT foam was developed as an adsorbing material. In this study, the performances of two gas-adsorbing materials—the synthesized CNT foam and commercial adsorbing material—with low-concentration ethane gas were compared using the developed μ-PC.

## 2. Materials and Methods

### 2.1. Principles and Requirements

The μ-PC captures trace (<100 ppb) VOC analytes from a large volume of standard gases and then thermally desorbs them into a much smaller volume; this increases their effective concentrations, which facilitates stable detection of the analytes. Therefore, a high-performance μ-PC should have (i) a sufficient adsorbent surface area for quantitative trapping of analytes from the gas samples; (ii) the ability to rapidly heat the adsorbent mass to the desired temperature (typically 250*–*300 °C) with a low thermal mass for quick heating/cooling and sufficiently good thermal isolation during heating, and (iii) a small pressure drop for low power/energy consumption during its preconcentration operation.

### 2.2. Materials

CNTs have recently been utilized as adsorbent agents of VOCs owing to their chemical and thermal stability, highly-specific surface area, and large affinity to nonpolar compounds [40,41,42,43]. In particular, since single-walled CNTs (SWCNT) exhibit larger aspect ratios and higher effective surface areas, they are more suitable as adsorbing materials than multi-walled CNTs (MWCNT) [44,45]. In this work, CNT foam was synthesized and employed as a gas-adsorbing material; the foam had a large gas adsorption capacity and low-pressure drop because of its high specific surface area and high porosity, respectively. Given its excellent performance, the CNT foam is assumed to have great potential as a gas-adsorbing material for gas preconcentration applications.

#### Synthesis Method for CNT Foam

**Figure 2:**
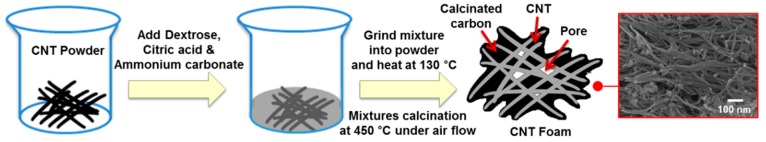
Schematic diagram showing the CNT foam synthesis procedure. CNT foam was obtained from mixture calcination in airflow. The inset shows the SEM micrograph of the synthesized CNT foam showing the presence of many voids and calcinated carbon within the entangled CNT foam.

SWCNT (>90% purity) was supplied in the form of nanopowder (SA-210, Nano Solution Inc., Hwaseong, Korea). Dextrose, citric acid, and ammonium carbonate were purchased from Daejung Chemicals & Metals Co., Ltd. All other solvents were used as received. CNT foam was synthesized by a chemical mixing and decomposition method. Dextrose and ammonium carbonate were used as a carbon source and pore-forming agent, respectively, to synthesize CNT foam. CNT powder with a mass of 250 mg was mixed with 500 mg of dextrose and 700 mg of citric acid at room temperature. Ammonium carbonate with a mass of 500 mg was then added to the above mixture and stirred until a homogeneous black powder was obtained. The obtained powder mixture was heated in an oven at 130 °C for 5 h. Finally, the CNT foam was prepared by decomposing the CNT foam precursor at 450 °C for 3 h in airflow. During calcination at a high temperature, the dextrose and citric acid were decomposed into carbon, while the ammonium carbonate was decomposed into gaseous products, leaving behind many pores. The specific surface area and mean pore diameter of the synthesized CNT foam were measured to be 403.97 m^2^/g and 7.18 nm, respectively, using the Brunauer, Emmett and Teller gas sorption method. Figure 2 shows the synthesis processes and photographs of CNT foam used for the adsorption of low-concentration gases introduced into a preconcentrator chip and its SEM micrograph.

### 2.3. Microfabrication

**Figure 3:**
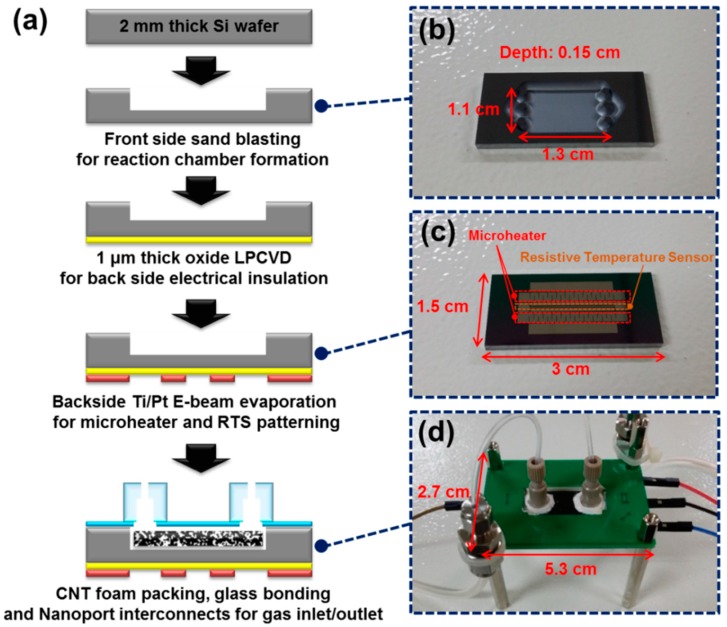
Micro gas preconcentrator (μ-PC) module: (**a**) schematic diagram showing the fabrication process of the μ-PC module; (**b**) the front-side view of the fabricated μ-PC chip showing the gas adsorption chamber with an array of posts for packing adsorbing material; (**c**) back-side view of the fabricated μ-PC chip showing the platinum micro-heater and resistive temperature sensor (RTS) for thermally desorbing volatile organic compound (VOC) gas molecules from the CNT foam and temperature control, respectively; (**d**) the packaged μ-PC module with fluidic port interconnections, mounted and wire bonded to a printed circuit board (PCB) used for the preconcentration and thermal desorption (TD) of trace-level ethane gases.

Figure 3a shows the fabrication flow of a μ-PC module filled with CNT foam. The μ-PC chamber and micro-heater layer were fabricated using the conventional MEMS fabrication process from a p-doped <100> Si wafer (double-side-polished) substrate with a thickness of 2 mm. The front side of the substrate was etched approximately 1.5 mm deep using a sand blasting technique to form the μ-PC chamber with an array of posts for packing the adsorbing material. The chamber size was 13 mm (L) × 11 mm (W), as shown in Figure 3b. To prevent an applied heating current from flowing through the entire μ-PC chip, and to concentrate only on the micro-heaters, a 1 μm-thick silicon oxide layer was deposited on the backside of the substrate with a low-pressure chemical vapor deposition (LPCVD) process to serve as an electric insulator. A 20 nm-thick titanium layer used for the adhesion and a 200 nm-thick platinum layer were deposited on the silicon oxide layer by electron beam evaporation and patterned by the lift-off process. Consequently, the micro-heater and resistive temperature sensor (RTS) were constructed on the backside of the substrate to thermally desorb VOC gas molecules from the CNT foam. The resistances of the micro-heater and the RTS were 32 and 60 Ω, respectively, as shown in Figure 3c. The CNT foam with a volume of approximately 214.5 mm^3^ was loaded in the μ-PC chamber; visual inspection confirmed that the CNT foam successfully filled the entire space of the chamber. The μ-PC chip was then sealed with 0.5 mm-thick Pyrex glass with two holes, each with a diameter of 1 mm, for the inlet/outlet channels by thermal bonding using a small amount of ceramic adhesive (Ultra-Temp 516, AREMCO Products Inc., Valley Cottage, NY, USA). This bond was treated at 350 °C for 6 h in an electric furnace (KSL-1100X, MTI Corp., Richmond, CA, USA). The μ-PC chip was attached to a printed circuit board (PCB) and fixed on the four edges of the inside square cavity using the same adhesive. The on-chip heater and RTS on the backside layer were wire bonded to electrical pads on the PCB for temperature control. Inlet and outlet fluidic channels were fashioned with a set of Nanoport Assemblies (N-333, IDEX Corp., Lake Forest, IL, USA), which were bonded to the inlet/outlet holes on the front side of the Pyrex glass using epoxy (Duralco 4703, Contronics Corp., Brooklyn, NY, USA). The outlet port was then inserted in the commercial gas chromatography-flame ionization detector (GC-FID). Figure 3d shows the fabricated μ-PC module with dimensions of 5.3 cm (L) × 2.7 cm (W).

### 2.4. Experimental Setup and Performance Test

#### 2.4.1. Pressure-Drop Test of Gas-Adsorbing Materials

The low-pressure drop of a gas preconcentrator is an important factor for low power consumption in a portable preconcentration system. When nitrogen gas passed through the adsorbing materials, the pressure drop was measured using a differential pressure gage (CP-100, KIMO Instruments, Chevry-Cossigny, France) connected by 1/16 inch Union Tee (Swagelok, Solon, OH, USA) to the inlet and outlet of the preconcentrator. This facilitated the comparison of the pressure drop performance between our synthesized CNT foam and the commercial gas-adsorbing material (carbon molecular sieves of Carbosieve S-III product, Sigma-Aldrich, St. Louis, MO, USA). The commercial carbon molecular sieves are the porous carbon skeletal framework that remains after the pyrolysis of a polymeric precursor. These particles are a granular-typed adsorbing material and are used for the adsorption of light alkanes such as C_2_H_6_–C_5_H_12_. The specific surface area and mean pore diameter of carbon molecular sieves are known to be 1000 m^2^/g and 1 nm, respectively [46].

#### 2.4.2. Preconcentration Test of the µ–PC Module

**Figure 4:**
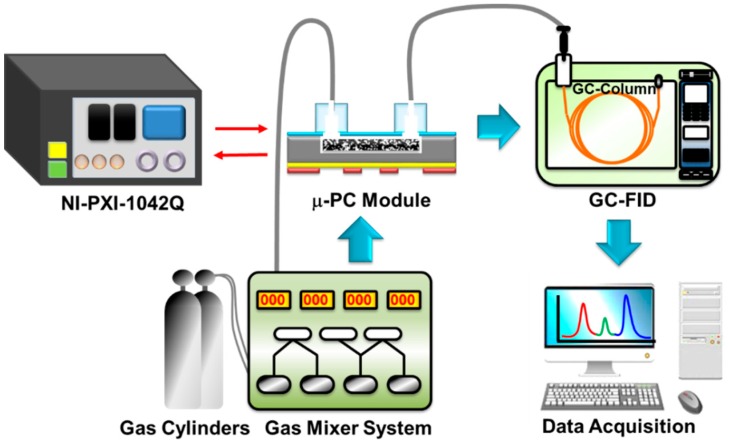
Experimental setup for the gas preconcentration performance tests consisting of the developed μ-PC module, a four-channel gas mixer system, a commercial GC-FID, and a system control unit. The μ-PC module was located upstream of the GC-FID and was directly connected to the inlet loop of the GC-FID using a fused silica capillary tube (360 μm-O.D., 150 μm-I.D.). The system control unit was responsible for the μ-PC module temperature via feedback control. The preconcentrated gas response signals were measured using the commercial GC-FID to ensure consistent testing parameters.

The experimental setup used for the gas preconcentration tests is shown in Figure 4. A gas mixer system consisting of mass flow controllers, solenoid valves, and on/off toggle switches was used to inject nitrogen (N_2_) carrier gas and standard ethane (C_2_H_6_) sample gas at a flow rate of 5 standard cubic centimeters per minute (sccm). The μ-PC module filled with CNT foam was located before the commercial GC-FID (YL 6100 GC, Young Lin Instrument, Anyang-si, South Korea) and was directly connected to the inlet loop of the GC-FID using a fused silica capillary tube (360 μm-outer dia., 150 μm-inner dia.) to ensure consistent testing parameters. This study was a preliminary test prior to the experiment of an actual breath gas sample, and the standard ethane sample gas at a concentration of 100 ppb was prepared as a low-concentration gas that was stably generated by dry nitrogen gas dilution using a gas mixer system. The sample gas was injected into the developed μ-PC module at a flow rate of 5 sccm at room temperature for 30 min, and then the ethane gas molecules were adsorbed onto the CNT foam surface within the μ-PC chip. Subsequent rapid heating of the embedded micro-heater at 25 °C/s up to 250 °C was conducted by supplying 4.8 W of electric power from a system control unit (PXI-1042Q, National Instrument, Austin, TX, USA) while the flow was stopped for 10 s. The temperature control by the μ-PC chip onboard RTS was used for the rapid TD process along with a programmed voltage supply algorithm. This process led to the desorption of the collected gas molecules into the column of the GC-FID, which used a nitrogen carrier gas at a flow rate of 0.1 sccm. The measurements using the GC-FID started at the same time as the desorption process, and the appearance time for the desorption peak of the preconcentrated gas followed the characteristics of a commercial GC column (GS-Gaspro, Agilent, Santa Clara, CA, USA). The same procedure was followed by the use of commercial carbon molecular sieves for the comparison of the preconcentration performance.

## 3. Results and Discussions

### 3.1. Pressure-Drop Test of Gas-Adsorbing Materials

**Figure 5:**
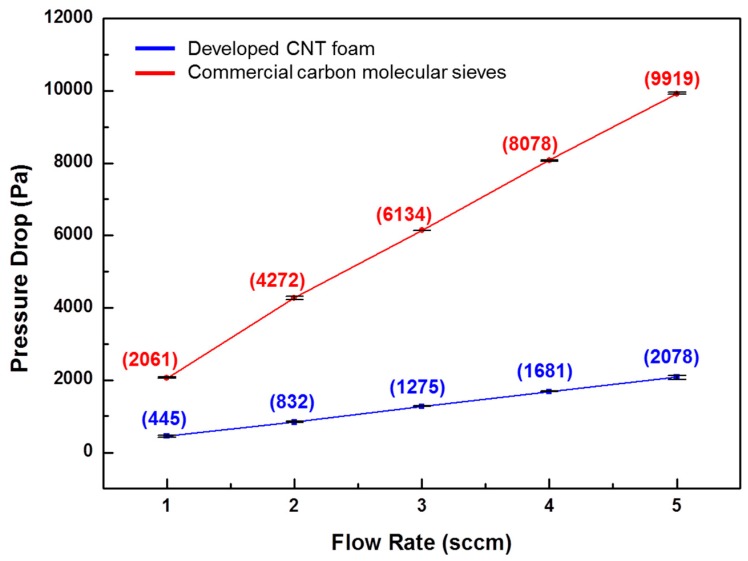
Comparison of pressure drop test results between the synthesized CNT foam and commercial carbon molecular sieves filled in μ-PC chips. The pressure drop test was performed using a differential pressure gage connected by Union Tee to the inlet and outlet of the μ-PC, with gas flow rates ranging from 1 to 5 sccm.

The pressure drop of the preconcentrator filled with two adsorbing materials was closely related to the flow rate. Figure 5 shows the results of the measured pressure drops for our synthesized CNT foam and commercial carbon molecular sieves. The pressure drop for the carbon molecular sieves rapidly increased from 2061 to 9919 Pa and was linearly dependent on the flow rate, which varied from 1 to 5 sccm. In comparison, when the synthesized CNT foam was tested under the same flow rate conditions a pressure drop ranging from 445 to 2078 Pa was yielded, which is a fivefold decrease relative to the commercial adsorbent. These results indicate that the use of the synthesized CNT foam as a gas-adsorbing material can significantly decrease the pressure drop in the gas preconcentration device.

### 3.2. Preconcentration Test of the µ–PC Module

**Figure 6:**
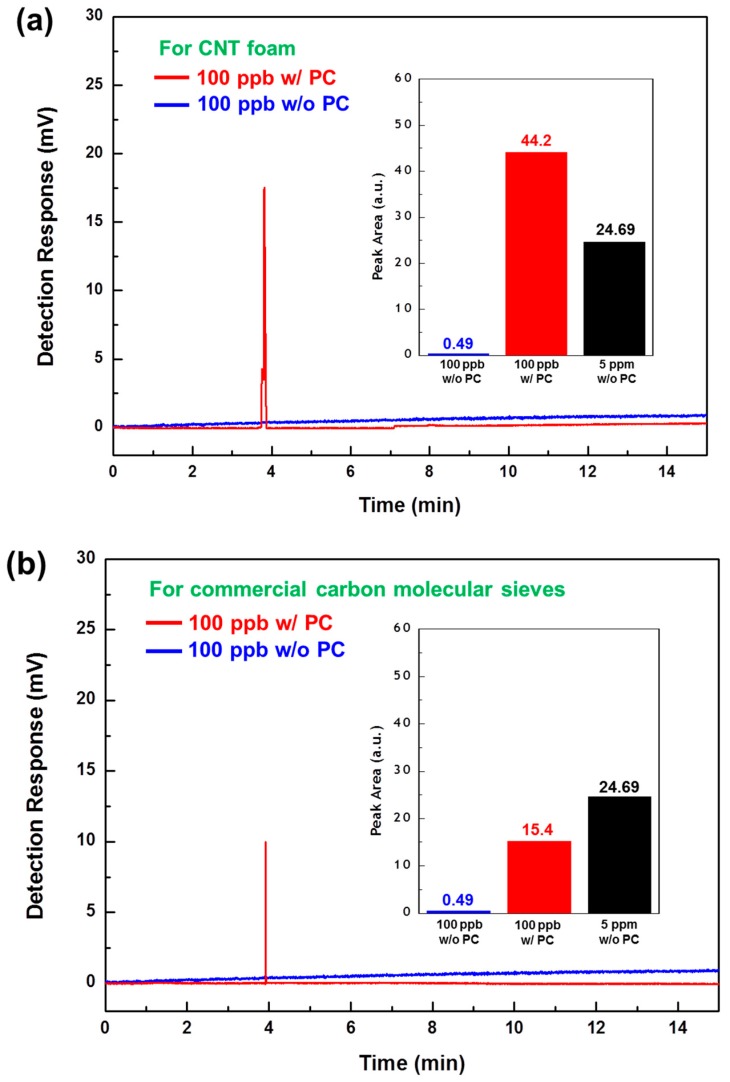
GC-FID gas detection peaks for ethane with and without the μ-PC module using: (**a**) synthesized CNT foam and (**b**) commercial carbon molecular sieves. Ethane gas diluted to 100 ppb concentration in nitrogen was preconcentrated at a flow rate of 5 sccm for 30 min before being thermally desorbed with temperature ramping at 25 °C/s to 250 °C within 10 s.

**Figure 7:**
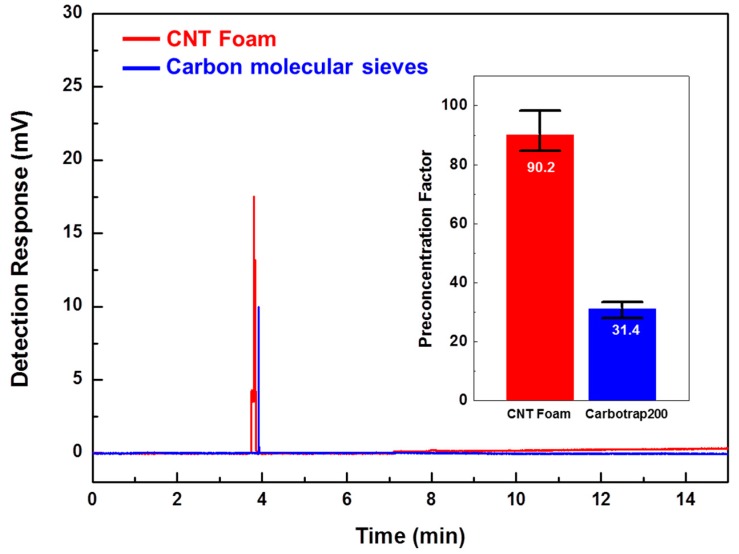
Comparison of gas preconcentration results and preconcentration factors (PFs) for ethane between the use of synthesized CNT foam and commercial carbon molecular sieves as gas-adsorbing materials.

The gas preconcentration efficiencies of two μ-PC modules filled with synthesized CNT foam and commercial adsorbing material, respectively, were evaluated using a commercial GC-FID. Generally, preconcentrators are characterized by a figure of merit known as the preconcentration factor (PF) [47]. The PF can be defined as the ratio of the peak area under the detection signal of the FID, with and without the presence of the preconcentrator. The preconcentration test of the developed μ-PC focused on a preliminary study of basic gas-adsorbing performance, and it was conducted with a standard ethane gas diluted to a concentration of 100 ppb in nitrogen at a flow rate of 5 sccm, with a 30-min adsorption process followed by a rapid TD process with temperature ramping at 25 °C/s to 250 °C within 10 s. The ethane gas sample was chosen as the target gas because it is a clinically-important biomarker for diseases, such as asthma and COPD, in exhaled breath. Figure 6 shows the GC-FID gas detection peaks with and without μ-PC modules using our synthesized CNT foam and commercial carbon molecular sieves, respectively. The peak area values for the CNT foam and carbon molecular sieves were 44.2 and 15.4 arbitrary units (AU), respectively. The value of 24.69 AU for standard ethane at a concentration of 5 ppm without preconcentration was used to compare each peak area value. A low detection response value of 0.49 AU was obtained for ethane at a concentration of 100 ppb without preconcentration, as shown in Figure 6a,b. The PFs for ethane gas using the CNT foam and carbon molecular sieves were 90.2 and 31.4, respectively. Thus, the gas preconcentration efficiency of our synthesized CNT foam was approximately three times higher than that of the commercial carbon molecular sieves, as shown in Figure 7.

## 4. Conclusions

In this study, a μ-PC module filled with the synthesized CNT foam was developed for the preconcentration of trace-level ethane gas, which can be used as a biomarker for medical conditions, such as asthma and COPD, in exhaled breath. The CNT foam was synthesized and used as a gas-adsorbing material for ethane adsorption. CNT foam possesses a large gas adsorption capacity owing to its large surface to volume ratio, low-pressure drop owing to its high porosity, and rapid TD owing to its high thermal conductivity. The use of commercial carbon molecular sieves as gas-adsorbing materials was compared with our synthesized CNT foam to examine the performance of the preconcentration. The μ-PC chip was designed and fabricated by conventional MEMS processes and its performance in terms of pressure drop and PF was tested using a differential pressure gage and commercial GC-FID, respectively. The test results showed that the pressure drop of the synthesized CNT foam was approximately five times lower than that of the carbon molecular sieves, and the gas preconcentration efficiency of μ-PC using the CNT foam was approximately three times higher than that of the carbon molecular sieves, according to the measured PF values. The novel miniaturized μ-PC module could not only overcome the detection limits of sensors but also lower the cost and power consumption of breath analysis systems. It also provides a focused sample injection technique to improve the chromatographic resolution of target gases. On the basis of these results, the developed μ-PC module can be considered to be an important component of an integrated micro gas analysis system. It is likely to be effective for the analysis of low-concentration exhaled breath biomarkers as part of noninvasive medical diagnoses and monitoring.
